# Preoperative chemotherapy for colon cancer and short-term outcomes—a nationwide cohort study

**DOI:** 10.1007/s00384-025-05060-z

**Published:** 2026-01-09

**Authors:** M. Delorme, E. Agger, F. Jörgren, M. L. Lydrup, H. Hagman, P. Buchwald

**Affiliations:** 1 Department of Surgery, Skåne University Hospital, Lund University, 214 21 Malmö, Sweden; 2 Department of Surgery, Hospital of Helsingborg, Lund University, Helsingborg, Sweden; 3 Department of Oncology, Skåne University Hospital, Malmö, Sweden

**Keywords:** Preoperative chemotherapy, Neoadjuvant treatment, Colon cancer, Mortality, Morbidity, Short-term outcomes

## Abstract

**Purpose:**

High-risk colon cancer may benefit from preoperative chemotherapy (preCHT), but evidence on its short-term safety and outcome is limited. Population-based evidence before its incorporation into national guidelines is lacking.

**Methods:**

Patients with final weighted stage II–III colon cancer undergoing elective resection between 2007 and 2017 were identified in the Swedish Colorectal Cancer Registry. Patients planned for preCHT, irrespective of intention, were compared with those undergoing upfront surgery. Primary outcomes were 30- and 90-day mortality and 30-day major morbidity, defined as all medical and surgical complications classified as Clavien-Dindo (CD) ≥ 3 grade. Subgroup analyses examined cT4 disease, and multivariable logistic regression was performed.

**Results:**

Among 20,185 eligible patients, 299 (1.5%) received preCHT. Postoperative mortality was comparable (1.7% vs. 1.7%, *p* = 1.00 at 30 days and 3.0% vs. 2.8%, *p* = 0.82 at 90 days). Overall and surgical postoperative morbidity (CD ≥ 3) was higher in the preCHT group (34.1 vs. 25.0%, *p* < 0.001 and 17.4% vs. 13.1%, *p* < 0.001), rates of anastomotic leakage were similar (3.3% vs. 3.6%, *p* = 0.85). Compared to upfront surgery, the preCHT group was more likely to undergo multivisceral resections (53.9% vs. 13.6%, *p* < 0.001), with a higher rate of R1 resections (6.4% vs. 3.2%, *p* < 0.001), reflecting more advanced disease (cT4: 59.5% vs. 10.5%, *p* < 0.001; cN1-2: 54.9% vs. 28.6%, *p* < 0.001). In the cT4 subgroup, short-term outcomes were comparable, and regression analyses found no independent association between preCHT and mortality or major morbidity.

**Conclusion:**

PreCHT appeared feasible in cT4N0-2M0 colon cancer, with short-term outcomes comparable to upfront surgery despite more advanced primary tumour and greater surgical extent.

## Introduction

Surgical resection is the conventional treatment of choice in colon cancer without distant metastases [1]. In high-risk patients, adjuvant chemotherapy is often recommended given the increased survival rate compared to surgery alone, attributed to its efficacy in addressing micro-metastasis [1, 2]. However, postoperative complications may delay succeeding treatment and may initiate a pathway for microscopic residual disease [3]. Local and/or systemic recurrence remains a concern, despite advances in adjuvant chemotherapy regimens and refinements in surgical techniques such as complete mesocolic excision [1, 4]. A recent population-based study using data from the Swedish Colorectal Cancer Registry (SCRCR) demonstrated a continued risk of recurrence following radical resection in stage II and III disease, suggesting the need to optimize treatment for high-risk tumours [5].

In patients with locally advanced and initially unresectable tumours, preoperative conversion treatment with chemotherapy and/or targeted therapy may be required to reduce local tumour involvement of adjacent organs and thereby improve R0 resection rates. While preoperative chemotherapy (preCHT) has been shown to be beneficial in gastroesophageal and rectal cancers, however, its clinical relevance of neoadjuvant treatment in high-risk resectable colon cancer is currently discussed and requires further evaluation [6–11]. Potential perioperative benefits of preCHT include improved radical resection rates, reduced tumour cell spillage, histopathologic downstaging, and, in some cases, complete pathological response [6, 12]. Recent randomized trials indicate that preCHT can be administered without increasing perioperative mortality or morbidity and that neoadjuvant oxaliplatin-based chemotherapy may reduce recurrence risk in selected patients with high-risk colon cancer [7, 13–15].

The current study aimed to evaluate short-term mortality and morbidity in a national, population-based cohort of patients with non-metastatic colon cancer who were for recommended preoperative antitumoral systemic treatment followed by surgical resection.

## Materials and methods

### Database description

Data was prospectively collected between 2007 and 2017 and retrospectively analyzed using SCRCR. The registry includes the majority of Swedish patients diagnosed with colon adenocarcinoma since 2007 and provides comprehensive information on patient demographics, tumour characteristics, treatment, short-term outcomes, and survival [16]. With more than 98% coverage, the SCRCR demonstrates high completeness and reliability, featuring a low proportion of missing data and strong internal and external validity for key variables [16]. Data from the SCRCR was extracted in April 2022, and the mortality information was obtained from the Swedish Cause of Death Registry.

### Data collection

The demographics analyzed were age, sex, body mass index (BMI), and American Society of Anesthesiologists (ASA) physical status classification. Clinical variables included clinical TNM-stage at diagnosis, primary tumour location, time between diagnosis and surgery, preCHT planned yes/no (regardless of treatment completion or intention), other preoperative interventions (preoperative stoma, stent, or both), and various surgical parameters (including surgical approach, multivisceral resection, tumour or colon perforation, blood loss, and stoma formation). Detailed information on systemic therapy regimens or treatment length was partly unavailable and therefore not retrieved from the SCRCR registry. Pathological variables assessed included microscopic radicality (R0/R1), occurrence of mucinous carcinoma, pathological T- and N-stage, and lymphovascular and perineural invasion, as well as microscopic tumour invasion into adjacent organs. A substantial proportion of histopathological data on tumour invasion into adjacent organs was missing in the registry, and these cases were treated as unknown rather than as absence of invasion. Primary outcome measures focused on 30-day postoperative morbidity (complications requiring medical or surgical intervention categorized as Clavien-Dindo ≥ 3 (CD ≥ 3), and mortality rates within 30- and 90-days postoperatively. Secondary outcome included surgical radicality.

Final weighted stage was defined according to the SCRCR annual report, Swedish national guidelines, and the Union for International Cancer Control TNM Classification of Malignant Tumours, 8th edition. T- and N-stage were primarily based on pathological assessment (pTN); if unavailable, clinical or radiological data (cTN) was used. M-stage was derived from the surgeon’s 30-day report, and if missing, from clinical or radiological data (cM), and finally from pathology (pM).

### Patient selection

Patients were categorized into two groups: those who were planned for preCHT followed by surgical resection and those who underwent upfront surgery without preoperative systemic treatment. The preCHT group included patients with high-risk resectable colon cancer and those requiring downstaging i.e. conversion therapy with chemotherapy and/or targeted therapy [7]. Between 2007 and 2017, neoadjuvant chemotherapy was not included in the Swedish national treatment guidelines; here, preCHT refers to systemic therapy given preoperatively in accordance with recommendations of that time, but outside standardized strategies.

Inclusion criteria were as follows: (1) adenocarcinoma in the colon—among patients with synchronous colon tumours, only the most advanced tumour was included; (2) final weighted tumour stage II and III, as defined above; and (3) elective surgical resection with curative intent. From the initially identified cohort, the following were excluded: (1) final weighted tumour stage I and IV, or unknown stage; (2) unplanned/emergent surgery; (3) patients who did not undergo tumour resection surgery e.g. local excision, polypectomy, or only laparotomy; (4) patients with synchronous rectal cancer, defined as a diagnosis within ± 6 months of the colon cancer diagnosis; (5) patients treated with preoperative radiotherapy; and (6) preCHT patients who underwent surgery within 60 days of diagnosis, based on the assumption that an oncological preoperative systemic treatment period of at least 6–8 weeks, followed by surgery, could not have been completed within that timeframe.

Within the overall cohort, the subgroup of patients with cT4N0–2M0 tumours was analysed separately.

### Statistical analysis

Study variables are presented as median with interquartile range (IQR) or as counts with corresponding percentages. Chi-square test or Fisher’s exact probability method was used to compare categorical data between the two groups. Continuous variables were analyzed using the Mann–Whitney *U* test.

Multivariable logistic regression was used to adjust for baseline differences, with postoperative complications as the dependent outcomes. Separate models were fitted for the whole cohort and the cT4 subgroup. Covariates included gender, age, ASA classification, BMI, tumour location, year of diagnosis, surgical approach, final weighted TNM stage. Model fit was assessed using the omnibus test (*p* < 0.05). To explore effect modification by tumour extent, identical analyses were performed in the cT4 subgroup. Upfront surgery served as the reference category. Missing values were left as missing to reflect real-world registry data. They were included in descriptive analyses but excluded from the multivariable regression models. A *p-*value of < 0.05 was considered statistically significant. All statistical analyses were carried out in IBM SPSS for Windows (version 28, IBM Corp, Armonk, NY, United States).

## Results

### General patient characteristics and demographics

**Fig. 1:**
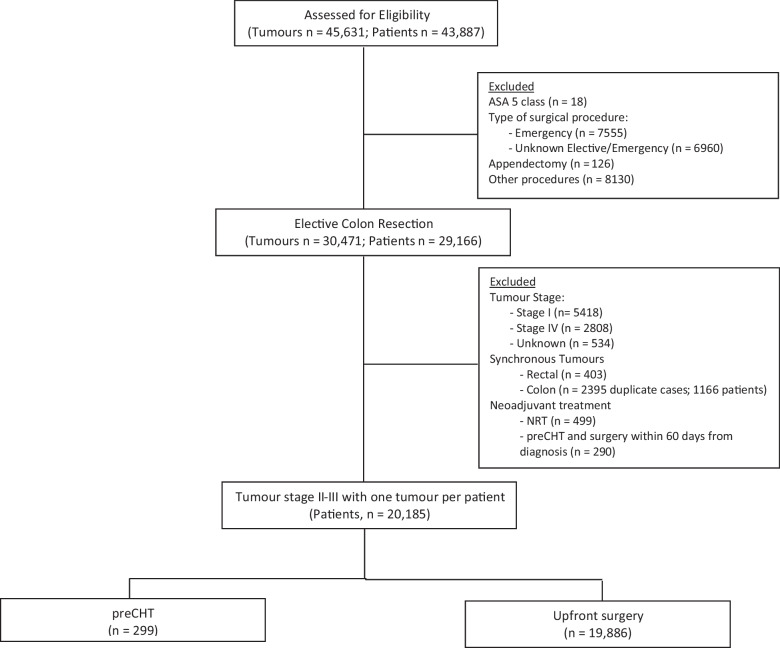
Study flow chart. preCHT, preoperative chemotherapy; NRT, neoadjuvant chemoradiotherapy

Of the 43,887 patients assessed for eligibility, 20,185 with final weighted stage II–III colon cancer who underwent elective resection were included in the analysis (Fig. 1). As presented in Table 1, only 1.5% received preCHT, while the rest of the cohort underwent upfront surgery. Patients in the preCHT group were younger, more often males, and more likely to have cT4 tumours, node-positive, and left-sided tumour location than the upfront surgery group. A preoperative MDT discussion was also more frequently performed before preCHT was recommended.

**Table 1:** Baseline patient characteristics in the overall cohort and cT4 subgroup

Variable	All patients (*n* = 20,185)		*p*	cT4 subgroup (*n* = 2259)		*p*
	preCHT (*n* = 299, 1.5%)	Upfront surgery (*n* = 19,886, 98.5%)		preCHT (*n* = 178, 7.9%)	Upfront surgery (*n* = 2081, 92.1%)	
Gender,* n *(%)			** < *0.001***			** < *0.001***
Male	167 (55.9)*	9 681 (48.7)		95 (53.4)*	911 (43.8)	
Female	132 (44.2)	10 205 (51.3)*		83 (46.3)	1 170 (56.2)*	
Age, median (IQR)	66 (15)	74 (14)*	** < *0.001***	65.5 (15)	73 (14)*	** < *0.001***
ASA classification, *n *(%)			0.17			0.46
ASA 1–2	216 (72.2)	13 179 (66.3)		131 (88.2)	1 428 (68.6)	
ASA 3	76 (25.4)	5 864 (29.5)		42 (23.6)	575 (27.6)	
ASA 4	5 (1.7)	479 (2.4)		4 (2.3)	43 (2.1)	
Missing	2 (0.7)	364 (1.8)		1 (0.6)	35 (1.7)	
BMI, *n* (%)			** < *0.001***			** < *0.001***
< 30	252 (84.3)*	15 223 (76.6)		157 (88.2)*	1 681 (80.8)	
≥ 30	33 (11.4)	3 143 (15.8)*		14 (7.8)	273 (13.1)*	
Missing	14 (4.7)	1 520 (7.6)		7 (3.9)	127 (6.1)	
Tumour location, *n *(%)			** < *0.001***			** < *0.001***
Right colon^a^	108 (36.1)	10 099 (50.8)*		64 (36.0)	1 043 (50.1)*	
Transverse	23 (7.7)	1 880 (9.5)		11 (6.2)	211 (10.1)	
Left colon^b^	168 (56.2)*	7 898 (39.7)		103 (57.9)*	826 (39.7)	
Unknown	-	9 (0.1)		-	1 (0.1)	
Missing	-	-		-	-	
Year of diagnosis			0.07			** < *0.001***
2007–2010	84 (28.1)	6 833 (28.1)		49 (27.5)	491 (23.6)	
2011–2014	120 (40.1)	7 135 (35.9)		78 (43.8)*	743 (35.7)	
2015–2017	95 (31.8)	5 918 (29.8)		51 (28.7)	847 (40.7)*	
Clinical T-stage,* n *(%)			** < *0.001***			*-*
cT1-2	11 (3.7)	3 166 (15.9)*		-	-	
cT3	60 (20.1)	6 789 (34.1)*		-	-	
cT4	178 (59.5)*	2 081 (10.5)		178 (7.9)	2 081 (92.1)	
cTx	37 (12.4)	4 929 (24.8)		-	-	
Missing	13 (4.4)	2 921 (14.7)		-	-	
Clinical N-stage, *n *(%)			** < *0.001***			** < *0.001***
cN0	81 (27.1)	9 920 (49.9)*		33 (18.5)	654 (31.4)*	
cN1-2	164 (54.9)*	5 688 (28.6)		117 (65.7)*	1 225 (58.9)	
cNx	52 (17.4)	3 940 (19.8)		28 (15.7)*	200 (9.6)	
Missing	2 (0.7)	338 (1.7)		-	2 (0.1)	
Pretherapeutic MDT, *n *(%)			** < *0.001***			1.00
Yes	287 (96.0)*	16 881 (84.9)		178 (100.0)	2 080 (99.9)	
Missing	-	18 (0.1)		-	-	

All patients include final weighted stage II–III colon cancer; the cT4 subgroup includes only patients with clinical T4 tumours. *p*-values compare preCHT vs. upfront surgery. Abbreviations: *preCHT*, preoperative chemotherapy; *ASA*, American Society of Anesthesiologists; *MDT*, multidisciplinary team. ^a^Includes cecum, ascending colon, and hepatic flexure. ^b^Includes splenic flexure, descending colon, and sigmoid colon. *Indicates statistically significant difference between groups

In the cT4 subgroup, these trends largely persisted. PreCHT patients remained younger and more likely to present with left-sided and node-positive disease. However, MDT discussion was documented in almost all cT4 patients in both the preCHT and upfront surgery groups, indicating consistent multidisciplinary management of high-risk tumours. No clear trends were observed in the overall use of preCHT over time during the study period.

### Surgical characteristics

As shown in Table 2, preoperative interventions were more frequent in the preCHT group, including a higher rate of defunctioning stomas. Open surgery was the predominant approach in both groups, although minimally invasive surgery was more frequently used in patients undergoing upfront surgery.

**Table 2:** Surgical characteristics in overall cohort and cT4 subgroup

Variable	All patients (*n* = 20,185)		*p*	**cT4 subgroup** (*n* = 2259)		*p*
	preCHT (*n* = 299)	Upfront surgery (*n* = 19,886)		preCHT (*n* = 178)	Upfront surgery (*n* = 2081)	
Preoperative interventions, *n (%)*			** < *0.001***			1.00
Diverting stoma	123 (41.1)*	551 (2.8)		84 (47.2)	141 (6.8)	
Stent	5 (1.7)	107 (0.5)		4 (2.3)	8 (0.4)	
Diverting stoma + stent	2 (0.7)	4 (0.02)		-	1 (0.1)	
Surgical approach, *n* (%)			0.31			** < *0.001***
Open	274 (91.6)	16 126 (81.1)		173 (97.2)	1921 (92.4)	
MIS	18 (6.0)	3 033 (15.3)		3 (1.7)	111 (5.3)*	
Converted MIS	7 (2.3)	727 (3.7)		49 (2.4)	2 (1.1)	
Temporary stoma			** < *0.001***			** < *0.001***
Yes	59 (19.7)*	683 (3.4)		46 (25.8)*	194 (9.3)	
Missing	2 (0.7)	67 (0.3)		1 (0.6)	4 (0.2)	
Permanent stoma			** < *0.001***			** < *0.001***
Yes	66 (22.1)*	1 062 (5.3)		44 (24.7)*	242 (11.6)	
Missing	3 (1.0)	134 (0.7)		2 (1.2)	13 (0.6)	
Multivisceral resection, *n* (%)			** < *0.001***			** < *0.001***
Yes	161 (53.9)*	2 694 (13.6)		121 (68.0)*	879 (42.2)	
Bowel perforation, *n* (%)			0.24			0.88
Yes	7 (2.3)	311 (1.6)		6 (3.4)	74 (3.6)	
Missing	1 (0.3)	138 (0.7)		-	17 (0.8)	
Blood loss, median (IQR)	300 (700)*	100 (250)	** < *0.001***	400 (800)*	200 (325)	** < *0.001***
Time between diagnosis to surgery*, in days*, median (IQR)	148 (87)*	34 (24)	** < *0.001***	150 (64)*	34 (25)	** < *0.001***

All patients include final weighted stage II–III colon cancer; the cT4 subgroup includes only patients with clinical T4 tumours. *p*-values compare preCHT vs. upfront surgery

*preCHT* preoperative chemotherapy, *MIS* minimal invasive surgery

*Indicates statistically significant difference between groups

Multivisceral resections were also more common following preCHT (53.9% vs. 13.6%, *p* < 0.001), often involving adjacent pelvic or abdominal organs. Intraoperative blood loss was greater, and time to surgery was longer due to planned systemic treatment.

In the cT4 subgroup, these patterns remained. Stoma formation, including both temporary and permanent types, was more frequent following preCHT. Minimally invasive surgery was rarely used in either group, with open resection being the standard approach.

### Histopathological outcomes

PreCHT treated tumours were more often associated with adverse features, including pT4 stage, vascular invasion, perineural invasion, and R1 resections (Table 3). Complete pathological response (ypT0N0) was observed in only three patients.

**Table 3:** Histopathological outcomes in the overall cohort and cT4 subgroup

Variable	All patient**s** (*n* = 20,185)		*p*	cT4 subgroup (*n* = 2,259)		*p*
	preCHT (*n* = 299)	Upfront surgery (*n* = 19,886)		preCHT (*n* = 178)	Upfront surgery (*n* = 2081)	
Microscopic radicality, *n *(%)			** < *0.001***			0.93
R0	262 (87.6)	18 665 (93.9)		157 (88.2)	1 838 (88.3)	
R1	21 (7.0)*	709 (3.6)		11 (6.2)	145 (10.0)	
Microscopically unclear margin	11 (3.7)	423 (2.1)		7 (3.9)	80 (3.8)	
Missing	5 (1.7)	89 (0.5)		3 (1.7)	18 (0.9)	
Mucinous carcinoma*, n *(%)			0.58			0.73
Yes	63 (21.1)	3 863 (19.8)		37 (20.8)	474 (22.8)	
Unknown	14 (4.7)	1 143 (5.9)		6 (3.4)	86 (4.1)	
Missing	4 (1.3)	39 (0.2)		2 (1.1)	11 (0.5)	
Pathological T-stage, *n *(%)			** < *0.001***			** < *0.001***
pT0	3 (1.0)	-		1 (0.6)	-	
pT1	1 (0.3)	190 (1.0)		-	4 (0.2)	
pT2	3 (1.0)	734 (3.7)*		1 (0.6)	12 (0.6)	
pT3	142 (47.5)	14 794 (74.4)*		65 (36.5)	989 (47.5)*	
pT4	144 (48.2)*	4 131 (20.8)		105 (59.0)*	1 069 (51.4)	
pTx	3 (1.0)	13 (0.1)		3 (1.7)	-	
Missing	3 (1.0)	24 (0.1)		3 (1.7)	7 (0.3)	
Pathological, n-stage, *n *(%)			0.52			0.17
pN0	155 (51.8)	10 263 (52.6)		98 (55.1)	996 (47.9)	
pN1	82 (27.4)	5 910 (30.3)		45 (25.3)	611 (29.4)	
pN2	58 (19.4)	3 278 (16.8)		32 (18.0)	463 (22.3)	
pNX	1 (0.3)	39 (0.2)		1 (0.6)	4 (0.2)	
Missing	3 (1.0)	33 (0.2)		2 (1.1)	7 (0.3)	
Final weighted tumour-stage, *n *(%)			0.89			** < *0.001***
II	157 (52.5)	10 522 (52.8)		100 (56.2)*	1 003 (48.2)	
III	142 (47.5)	9 364 (47.1)		78 (43.8)	1 078 (51.8)*	
Vascular invasion,* n *(%)			** < *0.001***			0.24
Yes	96 (32.1)*	5 301 (26.7)		55 (30.9)	757 (36.4)	
Unknown	13 (4.4)	1 406 (7.1)*		7 (3.9)	107 (5.1)	
Missing	4 (1.3)	66 (0.3)		3 (1.7)	18 (0.9)	
Perineural invasion,* n *(%)			** < *0.001***			0.70
Yes	61 (20.4)*	2 668 (13.4)		37 (20.8)	397 (19.1)	
Unknown	31 (10.4)	2 779 (14.0)*		14 (7.9)	196 (9.4)	
Missing	5 (1.7)	65 (0.3)		4 (2.3)	16 (0.8)	
Other organ invasion,* n *(%)			** < *0.001***			** < *0.001***
Yes	79 (26.4)*	1 018 (5.1)		67 (37.6)*	485 (23.5)	
Missing	195 (65.2)	17 077 (85.9)		100 (56.2)	1 291 (62.0)	

All patients include final weighted stage II–III colon cancer; the cT4 subgroup includes only patients with clinical T4 tumours*. p*-values compare preCHT vs. upfront surgery. Abbreviations: preCHT, preoperative chemotherapy. *Indicates a statistically significant difference between groups

In the cT4 subgroup, margin status is comparable with fewer R1 resections after preCHT. Although pT4 tumours remained more common in the preCHT group, the final weighted stage was more often stage II than stage III, while the opposite was seen in the upfront surgery group. Other pathological features, including nodal status and invasion markers, did not differ. Reports of tumour invasion into adjacent organs were more frequent following preCHT; however, this variable had a high proportion of missing data.

### Postoperative mortality and morbidity

30- and 90-day postoperative mortality was low and comparable between groups (1.7% vs. 1.7%, *p* = 1.00; and 3.0% vs. 2.8%, *p* = 0.82, respectively) (Table 4). Serious complications (CD ≥ 3) were more frequent in the preCHT group, with higher rates of both overall and surgical morbidity. Cardiovascular and neurological events were also more common, though absolute numbers remained low.

**Table 4:** Postoperative mortality and morbidity in overall cohort and cT4 subgroup

Variable	All patients (*n* = 20,185)		*p*	cT4 subgroup (*n* = 2259)		*p*
	preCHT (*n* = 299)	Upfront surgery (*n* = 19,886)		preCHT (*n* = 178)	Upfront surgery (*n* = 2081)	
30-day mortality, *n *(%)	5 (1.7)	341 (1.7)	1.00	3 (1.7)	44 (2.1)	1.00
90-day mortality, *n *(%)	9 (3.0)	556 (2.8)	0.82	5 (2.8)	76 (3.7)	0.56
Any CD ≥ 3, *n* (%)			** < *0.001***			0.08
Yes	102 (34.1)*	4 961 (25.0)		66 (37.1)	639 (30.7)	
Missing	-	28 (0.1)		-	6 (0.1)	
Surgical complications, CD ≥ 3,* n *(%)			** < *0.001***			0.23
Yes	52 (17.4) *	2 612 (13.1)		31 (17.4)	294 (14.1)	
Missing	-	-		-	-	
Type of surgical complications, *n* (%)*ǂ*						
Anastomotic leakage	10 (3.3)	705 (3.6)	0.85	5 (2.8)	71 (3.4)	0.67
Stoma complication	2 (0.7)	50 (0.3)	0.16	2 (1.1)	10 (0.5)	0.26
Intraabdominal infection	13 (4.4)*	457 (2.3)	** < *0.001***	9 (5.1)	71 (3.4)	0.25
Wound infection	16 (5.4)	817 (4.1)	0.28	9 (5.1)	99 (4.8)	0.86
Organ perforation	1 (0.3)	39 (0.2)	0.59	1 (0.6)	7 (0.3)	0.63
Bleeding	1 (0.3)	215 (1.1)	0.21	1 (0.6)	16 (0.8)	0.76
Wound rupture	12 (4.0)	551 (2.8)	0.20	7 (3.9)	60 (2.9)	0.43
Type of medical complications, *n* (%)						
Cardiovascular	18 (6.0)*	722 (3.6)	** < *0.001***	9 (5.1)	103 (5.0)	0.95
Neurological	3 (1.0)*	64 (0.3)	** < *0.001***	1 (0.6)	6 (0.3)	0.53
Respiratory/pulmonary	1 (0.3)	71 (0.4)	0.95	-	16 (0.8)	0.24
Other	29 (9.7)*	1 202 (6.0)	** < *0.001***	21 (11.8)	170 (8.2)	0.09

All patients include final weighted stage II–III colon cancer; the cT4 subgroup includes only patients with clinical T4 tumours. *p*-values compare preCHT vs. upfront surgery. Abbreviations: *preCHT*, preoperative chemotherapy; *CD ≥ 3*, Clavien-Dindo grade 3 or higher. *ǂMay have more than one complication per patient.* *Indicates statistically significant difference between groups

In the cT4 subgroup, overall complication rates were similar, and no notable differences were observed in major morbidity, surgical events, or anastomotic leakage.

#### Regression analyses

Among patients with major complication (Any CD ≥ 3), preCHT was associated with increased risk of wound rupture (OR 1.93, 95% CI 1.11–3.36, *p* < 0.05), and a numerical trend toward cardiovascular events. In the cT4 subgroup, no clear associations were found between preCHT and postoperative mortality or morbidity; however, odds ratios for Any CD ≥ 3 and “other” complications indicated a possible excess risk that did not reach significance  (Tables 5 and 6).

**Table 5:** Multivariable logistic regression of complication types among patients with major morbidity (any CD > 3)

Outcome	OR (95% CI)	*p*
30-day mortality	0.85 (0.26—2.76)	0.79
90-day mortality	1.01 (0.39—2.58)	0.99
Any CD ≥ 3	0.77 (0.51—1.16)	0.21
Surgical complications CD ≥ 3	0.56 (0.29—1.09)	0.09
Anastomotic leakage	1.77 (0.41—7.57)	0.44
Stoma complication	1.24 (0.67—2.30)	0.50
Intraabdominal infection	0.82 (0.47—1.45)	0.50
Wound infection	1.36 (0.18—10.35)	0.77
Bleeding	1.27 (0.66—2.42)	0.47
Wound rupture	1.93 (1.11—3.36)	** < 0.05**
Cardiovascular	3.01 (0.90—10.08)	0.073
Neurological	0.83 (0.11—6.18)	0.86
Respiratory/pulmonary	1.36 (0.87—2.12)	0.18
Other	0.85 (0.26—2.76)	0.79

*CI*, confidence interval; *OR*, odds ratio. Ref. upfront surgery. Adjusted for age, gender, ASA classification, BMI, tumour location, year of diagnosis, surgical approach, and final weighted TNM stage

**Table 6:** Multivariable logistic regression of postoperative outcomes in the cT4 subgroup

Outcome	OR (95% CI)	*p*
30-day mortality	1.09 (0.25—4.79)	0.91
90-day mortality	1.24 (0.42—3.61)	0.70
Any CD ≥ 3	1.33 (0.95—1.86)	0.10
Surgical complications CD ≥ 3	1.24 (0.81—1.89)	0.31
Anastomotic leakage	0.90 (0.35—2.30)	0.82
Stoma complication	2.37 (0.47—11.86)	0.29
Intraabdominal infection	1.39 (0.66—2.90)	0.38
Wound infection	0.94 (0.46—1.93)	0.87
Organ perforation	1.56 (0.17—14.04)	0.69
Wound rupture	1.49 (0.65—3.42)	0.34
Cardiovascular	1.34 (0.62—2.90)	0.45
Neurological	4.12 (0.39—43.37)	0.24
Other	1.58 (0.96—2.61)	0.07

*CI*, confidence interval; *OR*, odds ratio. Ref. upfront surgery. Adjusted for age, gender, ASA classification, BMI, tumour location, year of diagnosis, surgical approach, and final weighted TNM stage

## Discussion

This nationwide, population-based cohort shows that before 2018, preCHT was mainly offered to a few selected patients with high-risk, non-metastatic resectable colon cancer, particularly cT4 disease.  Despite higher surgical complexity and frequent multivisceral resections, short-term outcomes for mortality and major morbidity after preCHT were comparable to upfront surgery in subgroup and adjusted analyses.

The present cohort was restricted to 2007–2017, before preliminary results from the FOxTROT trial were reflected in the Swedish national guidelines for colon cancer in 2021, to capture practice patterns in an era when preCHT was not implemented as a strict neoadjuvant treatment strategy for resectable cases. The randomised FOxTROT trial demonstrated that 6 weeks’ neoadjuvant oxaliplatin–fluoropyrimidine chemotherapy for resectable high-risk colon cancer improved 2-year disease control without increasing perioperative morbidity [7]. The Swedish participation in FOxTROT was limited to a single center in 2015–2016, indicating that most patients in our cohort received preCHT as local initiatives or conversion therapy rather than within a trial framework. Patients selected for preCHT were generally younger with lower ASA scores reflecting selection of those considered fit for systemic therapy, in line with other studies [7, 9, 17]. The high rate of defunctioning stomas reflects a manifested or impending obstruction, for which guidelines recommend decompression rather than emergency colectomy [7, 18]. Such patients were almost absent in published randomised trials, with only 1% having stoma in the FOxTROT trial and obstruction excluded in others [7, 14, 15].

Although overall morbidity was higher after preCHT, this reflected advanced disease and complex resections, as the subgroup and adjusted analyses showed no excess short-term mortality or morbidity. Cardiovascular events were slightly more frequent, likely reflecting comorbidity and perioperative risk rather than chemotherapy-specific effects, as fluoropyrimidine-associated cardiotoxicity typically occurs during treatment and affects only a small proportion of patients, often without lasting sequelae [19, 20]. Anastomotic leakage rates were similar, consistent with FOxTROT and preliminary data from the similar randomised Danish NeoCol trial [7, 15]. Absolute numbers were low, but wound rupture appeared more common after preCHT among patients with Any CD ≥ 3 in the regression model, while no clear association was seen in the cT4 subgroup. Randomised trials investigating the addition of EGFR-inhibitors to chemotherapy in the neoadjuvant setting have reported increased perioperative risks with the combination [7, 14]. In our cohort, EGFR-inhibiting antibodies may have been used alone or combined with chemotherapy to enhance response in inoperable, locally advanced RAS- and BRAF-wildtype tumours. Overall, our findings fall within the reported range of postoperative outcomes after preCHT, with mortality ranging from 0 to 17% and morbidity from 2 to 21% in other studies [7, 8, 12, 14, 21, 22].

Locally advanced colon tumours may invade or appear adherent to neighbouring organs, but only histopathology distinguishes true infiltration from inflammatory adherence related to tumour-associated inflammation. Suspected infiltration is therefore frequently managed with en bloc resection to achieve R0, although histopathology confirms malignant invasion in only about half of such cases [23]. In our cohort, histopathological confirmation of adjacent organ invasion was frequently missing, while reporting of other variables such as vascular or perineural infiltration was consistent and the reason for this discrepancy is unclear. More than half of the preCHT group underwent multivisceral resections, a procedure associated with high morbidity and mortality, yet short-term outcomes remained comparable to upfront surgery in both subgroup analyses and adjusted models [23]. Published randomised trials on neoadjuvant chemotherapy enrolled cT3 or cT4N0-2 colon tumours, while true cT4b with adjacent organ invasion is underrepresented; FOxTROT reported 5% to be cT4b, whereas the PROGIGE 22 and NeoCol trials did not provide separate data [7, 14, 24]. Registry-based studies add context, showing potential survival benefit confined to cT4b disease, but also frequent overstaging and inconsistent evidence of tumour downstaging [22, 25]. In these cohorts, preCHT appeared to be given mainly in cT4b cases with uncertain resectability, rather than to patients with pathologically confirmed T4b infiltration [22, 25]. By contrast, published reports on preoperative therapy in this setting have largely focused on chemoradiotherapy or radiotherapy, administered either concomitantly or sequentially, and almost exclusively in left-sided colon tumours [26, 27]. Despite the published literature ranging from chemoradiotherapy and radiotherapy series to randomised trials and registry studies on neoadjuvant chemotherapy, no study has clearly defined the role of preoperative systemic therapy in patients requiring multivisceral resections [7, 14, 22, 24–27].

The predominance of stage II tumours, despite higher rates of pT4 in our cohort, could reflect nodal or tumour downstaging, but misclassification due to registry error is also possible. Computed tomography is known to be unreliable for early-stage colon cancer, as shown by Wetterholm et al., where many cT1–2 cases proved to be pT3–4 at pathology, and by Korsbacke et al., who demonstrated only fair agreement between CT and histopathology together with inaccuracies in SCRCR registration [28, 29]. In this context, the few cT1–2 cases recorded in our preCHT group are therefore unlikely to represent true candidates for systemic therapy and are more likely to reflect misclassification, although downstaging after treatment cannot be entirely excluded.

Positive resection margins are an established adverse prognostic factor in colon cancer [30]. In the cT4 subgroup, R1 resections were not increased and appeared lower after preCHT consistent with trial data [7]. This finding possibly reflects more frequent en bloc resections, where adherent organs are removed due to inflammatory reaction rather than true infiltration, or where downstaging reduced margin involvement.

While postoperative complications may adversely affect survival, early systemic therapy through preCHT offers the potential advantage of controlling micrometastatic disease at an earlier stage [7, 30]. In our registry-based national cohort, which included more advanced and surgically demanding tumours, possibly longer preCHT treatment periods, and other treatment regimens than modern neoadjuvant trial populations, short-term outcomes in the cT4N0-2 subgroup were comparable between preCHT and upfront surgery.

Given the descriptive design of this study, causal inference cannot be drawn, and findings should be interpreted as associations. The study was also limited by the low proportion of patients planned for preCHT, and no propensity score matching was feasible given marked baseline differences and the study scope. Instead, subgroup analyses and multivariable models were applied to address key confounders. Data quality relied on registered treatment intent at diagnosis and the timing of surgery, rather than confirmed administration of preCHT, meaning that some patients recorded as planned may not have received, completed, or tolerated the intended regimen. Although predefined exclusion criteria were applied, misclassification of exposure remains possible. Information on treatment strategies such as interruptions, delays, or regimen details was not captured, and mismatch repair status was not systematically available in the registry during the study period. Missing values were left unchanged, as they reflect real-world clinical registration. While this reduced sample size and statistical power, missing data was evenly distributed across treatment groups and is unlikely to have introduced systematic bias. Finally, the registered date of diagnosis in the SCRCR showed variability, with only 44% accuracy, mainly due to inconsistencies in definition, whether based on the date of diagnostic radiology, colonoscopy, pathology report, or the initial surgical consultation [16]. However, it is unlikely that having a more exact diagnostic date would have materially influenced our results or conclusion. The principal strength of this study lies in its use of a nationwide, population-based cancer registry with retrospective analysis of prospectively collected real-time data, thereby ensuring high external validity and comprehensive national coverage.

Future research should examine cohorts treated after the introduction of neoadjuvant oxaliplatin-based chemotherapy for selected high-risk colon cancer into Swedish national guidelines in 2021. Linking both registry and medical records will be essential to capture treatment regimens, molecular markers, and long-term oncologic outcomes to refine patient selection for this approach.

## Conclusion

In Sweden, preoperative systemic antitumoral therapy for stage II–III colon cancer was very rare between 2007 and 2017. Among the 1.5% of patients recommended for such treatment, cT4 tumours and multivisceral resections were more common. However, in cT4N0–2 patients, short-term surgical outcomes including mortality did not differ between those planned for preoperative therapy and those undergoing upfront surgery. This nationwide register-based retrospective study therefore suggests that a preoperative systemic therapy approach appears safe in patients having undergone surgery for high-risk, non-metastatic colon cancer.

## Data Availability

Access to individual-level data can be provided for research purposes but is restricted by Swedish confidentiality regulations and privacy laws. The data is therefore not publicly available but may be obtained after ethical approval and permission from relevant authorities.

## Declarations

### Prior submission

The abstract has been submitted for presentation at the Swedish Surgical Week 2025 in Linköping, Sweden (19–23 August 2025).
